# Evolution of the Local Structure in the Sol–Gel Synthesis of Fe_3_C Nanostructures

**DOI:** 10.1021/acs.inorgchem.0c03692

**Published:** 2021-05-04

**Authors:** Matthew S. Chambers, Dean S. Keeble, Dean Fletcher, Joseph A. Hriljac, Zoe Schnepp

**Affiliations:** † School of Chemistry, University of Birmingham, Birmingham B152TT, U.K.; ‡ Diamond Light Source, Harwell Science and Innovation Campus, Didcot OX11 0DE, U.K.

## Abstract

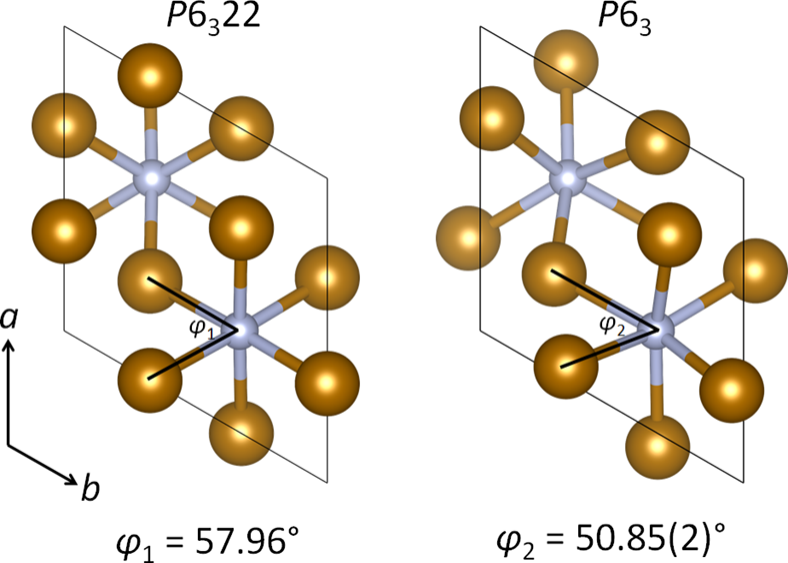

The sol–gel synthesis of iron carbide (Fe_3_C) nanoparticles proceeds through multiple intermediate crystalline phases, including iron oxide (FeO*_x_*) and iron nitride (Fe_3_N). The control of particle size is challenging, and most methods produce polydisperse Fe_3_C nanoparticles of 20–100 nm in diameter. Given the wide range of applications of Fe_3_C nanoparticles, it is essential that we understand the evolution of the system during the synthesis. Here, we report an *in situ* synchrotron total scattering study of the formation of Fe_3_C from gelatin and iron nitrate sol–gel precursors. A pair distribution function analysis reveals a dramatic increase in local ordering between 300 and 350 °C, indicating rapid nucleation and growth of iron oxide nanoparticles. The oxide intermediate remains stable until the emergence of Fe_3_N at 600 °C. Structural refinement of the high-temperature data revealed local distortion of the NFe_6_ octahedra, resulting in a change in the twist angle suggestive of a carbonitride intermediate. This work demonstrates the importance of intermediate phases in controlling the particle size of a sol–gel product. It is also, to the best of our knowledge, the first example of *in situ* total scattering analysis of a sol–gel system.

## Abstract 2

*In situ* total scattering analysis has uncovered the importance of iron oxide as an intermediate phase in the synthesis of Fe_3_C nanoparticles from sol−gel precursors. A sudden increase in order is observed between 300 and 350 °C, associated with the crystallization of iron oxide nanoparticles. The local structure of the Fe_3_N intermediate is also investigated.

## 1 Introduction

**Figure 1:**
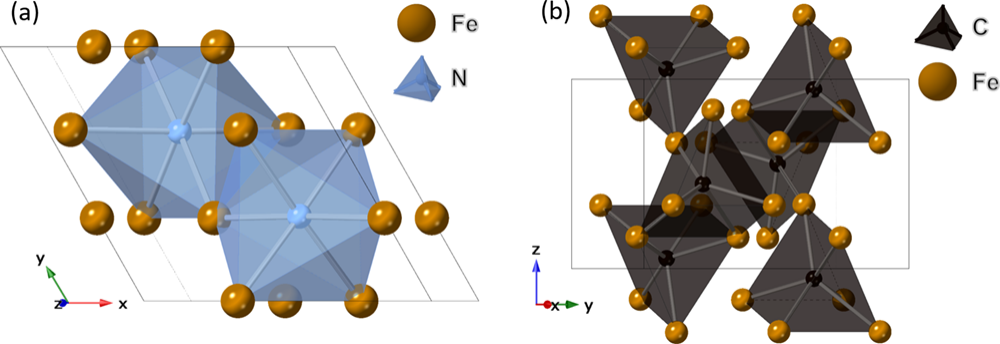
Crystal structures of (a) ε-Fe_3_N and (b) θ-Fe_3_C.

Iron forms a range of interstitial compounds with carbon and nitrogen, including ε-Fe_3_N and θ-Fe_3_C (Figure 1). These have been widely studied due to their importance in steel but are now receiving renewed attention for their potential as catalysts. Iron nitrides and carbides have been used as catalysts in the Fisher–Tropsch process,^1,2^ oxygen reduction reaction,^3^ and ammonia decomposition.^4^ Most recently, iron carbides and nitrides are being pursued due to their potential to replace rare and costly precious metals such as Pt in applications such as fuel cells.^5^ Additionally, θ-Fe_3_C (Fe_3_C) and ε-Fe_3_N (Fe_3_N) have interesting magnetic properties and uses in biomedical applications.^6−8^

In order to fully exploit the potential of Fe_3_N and Fe_3_C, it is important to have controlled routes to nanoparticles of these materials.^7^ Various routes have been proposed to achieve this goal, including laser ablation, ammonolysis of iron oxide nanoparticles, nanocasting,^9^ solvothermal synthesis^8,10^ and sol–gel chemistry. Sol–gel chemistry has the advantage of being relatively simple both in terms of the precursors and processing. In general, sol–gel synthesis of Fe_3_N or Fe_3_C nanoparticles is achieved by mixing aqueous iron salts (e.g., nitrate and acetate) with organic molecules such as urea^11^ or gelatin^12^ as well as with CTAB and melamine.^7^ The resulting “gel” is dried and pyrolyzed in an inert atmosphere to produce nanoparticles of the required product. While sol–gel chemistry is simple and scalable, it is difficult to achieve significant control over the particle size. It is also difficult to isolate pure nitride or carbide phases and small changes in experimental conditions can have a large effect on the product composition (Fe_3_N/Fe_3_C/Fe).^13^ In order to maximize the beneficial catalytic properties of iron nitrides and carbides and fully explore their potential, it is essential to gain a better understanding of how they are formed.

**Scheme 1:**
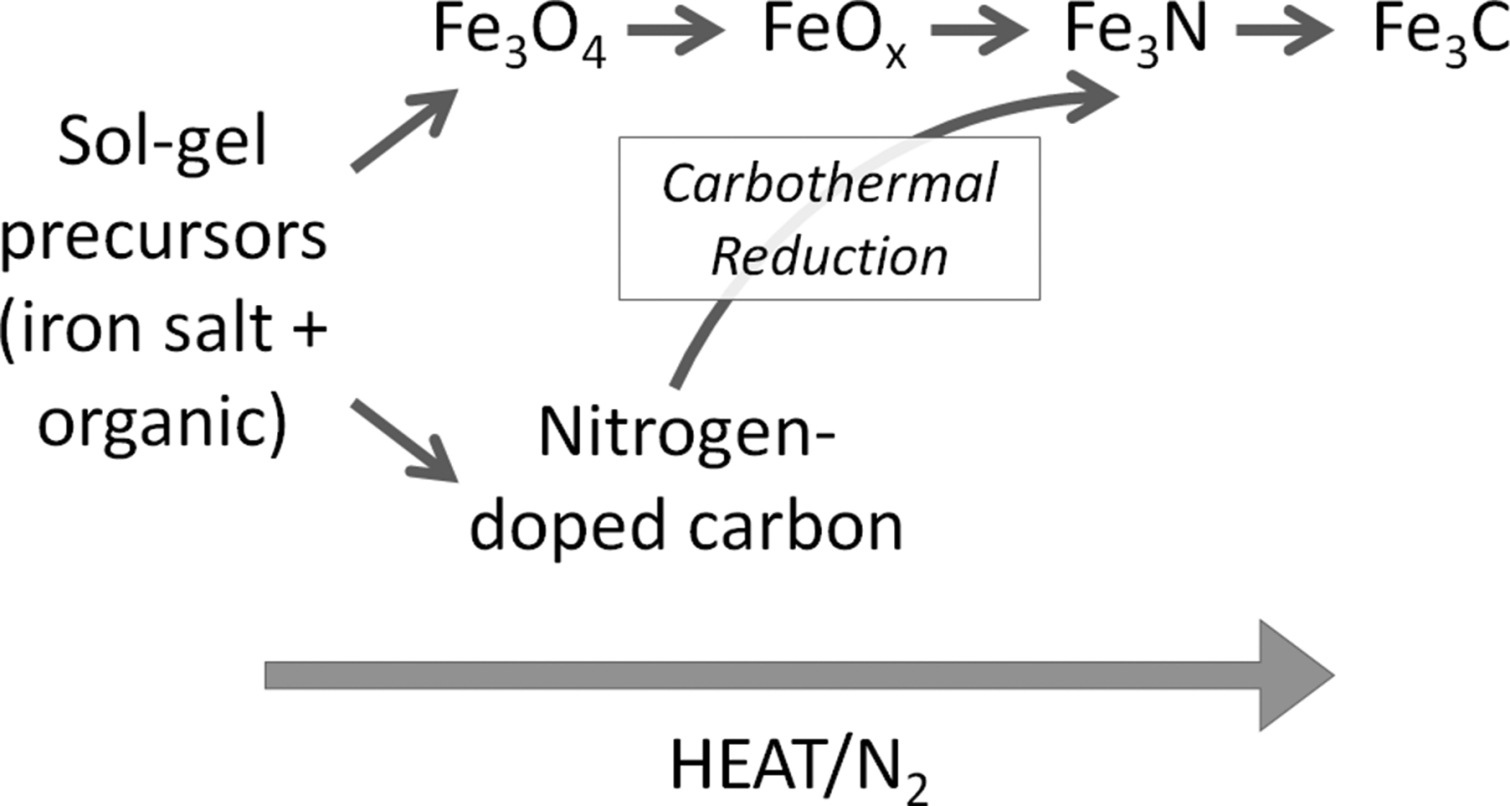
Proposed Reaction Mechanism for Fe_3_C Formation

In *in situ* synchrotron X-ray diffraction studies, we showed that the sol–gel route to Fe_3_C proceeds via several intermediates (Scheme 1).^12^ In a system involving gelatin and iron nitrate as precursors, the reaction was shown to proceed via an intermediate iron oxide phase. Significant peak broadening suggested that the particle diameter in this phase was very small (estimated at ∼3 nm). This is consistent with transmission electron microscopy (TEM) images that show iron oxide nanoparticles embedded in a carbon matrix. From 560 °C, sharp Fe_3_N peaks emerged and from 610 °C, sharp Fe_3_C peaks were observed, produced by carbothermal reduction and nitridation of the iron oxide intermediate by the surrounding nitrogen-doped carbon matrix. The Scherrer analysis indicated larger crystallite diameters of 30 nm (Fe_3_N) and 60 nm (Fe_3_C), again consistent with the TEM images. The Fe_3_N to Fe_3_C transition was believed to proceed via carbon diffusion into the nitride (forming a carbonitride intermediate), based on observations of a peak shift in the Fe_3_N phase.

We now report an *in situ* synchrotron total scattering study of the sol–gel synthesis of Fe_3_C. Total scattering and the pair distribution function (PDF) have been widely used *ex situ* to study the local order in crystalline and amorphous materials. They have also been used *in situ* to study nanoparticles formed in solvo/hydrothermal synthesis, showing the evolution of local and long-range structures.^14,15^ These systems, however, are comparatively simple as they involve only two or three precursor phases that evolve to a single phase suspended in a solvent. Total scattering has enormous potential to aid the understanding of sol–gel synthesis of materials. It offers information about local structural details that may be missed in Bragg scattering. It also allows us to examine the evolution of particle size and crystallinity at lower temperatures where no long-range order is present. The data in this study specifically demonstrate the very fast crystallization of the iron oxide intermediate during Fe_3_C synthesis. It also offers insight into the formation and structure of the Fe_3_N intermediate. To the best of our knowledge, this study is the first example of an *in situ* total scattering study of a sol–gel process. This is particularly significant as it shows that PDF analysis can be used to extract useful information from complex systems where there are multiple crystalline and amorphous components.

## 2 Experimental Procedure

### 2.1 Synthesis

The synthesis of the gelatin precursor was performed as described in the previous literature.^12^ A hot aqueous solution of gelatin (10%, w/w, 10 g, Sigma-Aldrich, G2500) with aqueous iron nitrate (10%, w/v, 20.2 mL, Fe(NO_3_)_3_·9H_2_O) formed a viscous orange gel. The orange gel was dried in air at 70 °C to produce a brittle orange-brown foam.

### 2.2 X-ray Total Scattering

X-ray total scattering data were collected using a wavelength of λ = 0.16167 Å. Samples of the orange-brown foam were ground and loaded into 1 mm diameter fused silica capillaries (with one end sealed) at the Diamond Light Source beamline I15-1. A hot air blower was used for the variable temperature experiments, and pure N_2_ was blown over the open end of the capillary to prevent oxidation of the sample. A temperature calibration was performed using a Si-Al_2_O_3_ standard.^16^ Data were collected from 150 to 400 °C in 50 °C increments and at 500 and 600 °C, with a heat rate of 10 °C min^–1^. Each data collection was 10 min in length. During heating, the samples underwent expansion due to the release of gases from gelatin decomposition, so the samples were occasionally repacked with a thin wire.

### 2.3 Rietveld Refinement

Rietveld refinements were performed using TOPAS v6.^17,18^ The starting models were derived from the following sources: FeO*_x_* (refined with the fixed stoichiometry of FeO) from Fjellvåg *et al*.,^19^ Fe_3_C from Wood *et al*.,^20^ and Fe_3_N from Jacobs *et al*.^21^ Backgrounds were described using sixth-order Chebyshev polynomials and with the scans of the empty fused silica capillaries collected at similar temperatures, where a refined scale factor was included. Peak shapes were described using the Thompson–Cox–Hastings pseudo-Voight function. Additionally, a zero-point parameter was refined. A range of 1.5 ≤ 2θ ≤ 20° was used. Refinements were performed against Bragg scattering obtained from the total scattering experiments described above for temperatures of 350, 400, 450, 500, and 600 °C. Attempts to perform Rietveld refinements against the data for 200–300 °C were made, but it was found that the entirety of the Bragg scattering can be described by the empty capillary backgrounds (Figure S1, Supporting Information). As the sample at 200 °C showed no Bragg scattering, no Rietveld analysis was attempted at 150 °C.

### 2.4 Small-Box PDF Refinements

Small-box PDF refinements were performed using TOPAS v6.^22^ The PDF data were obtained using GudrunX^23^ version 5 to produce *D*(*r*) data (as defined by Keen).^24^ The *D*(*r*) data were produced using *Q*_max_ = 20 Å^–1^. A Lorch^25^ correction function was used to remove Fourier ripples generated from the limited *Q*_max_. The broadening power of the function was set to 0.03 Å. For the refinement performed against the 600 °C data, the same phases used in the Rietveld refinement were included in the PDF refinements. Additionally, two amorphous carbon phases were modeled using graphite (starting model obtained from Trucano and Chen)^26^ for *sp*^2^ carbon and diamond (starting model obtained from Yamanaka and Morimoto)^27^ to model *sp*^3^ carbon. A function available in TOPAS^22^ that removes correlations at ranges of *r* = 5 Å was applied to these phases to model them as amorphous. Additionally, a further Fe_3_N phase with symmetry lowered from *P*6_3_22 to *P*6_3_ was included. The *P*6_3_ phase was limited to contributing to the PDF at *r* <4.1 Å, while *P*6_3_22 was limited to contributing at *r* >4.1 Å to simulate local ordering.

## 3 Results and Discussion

### 3.1 Rietveld Analysis

**Figure 2:**
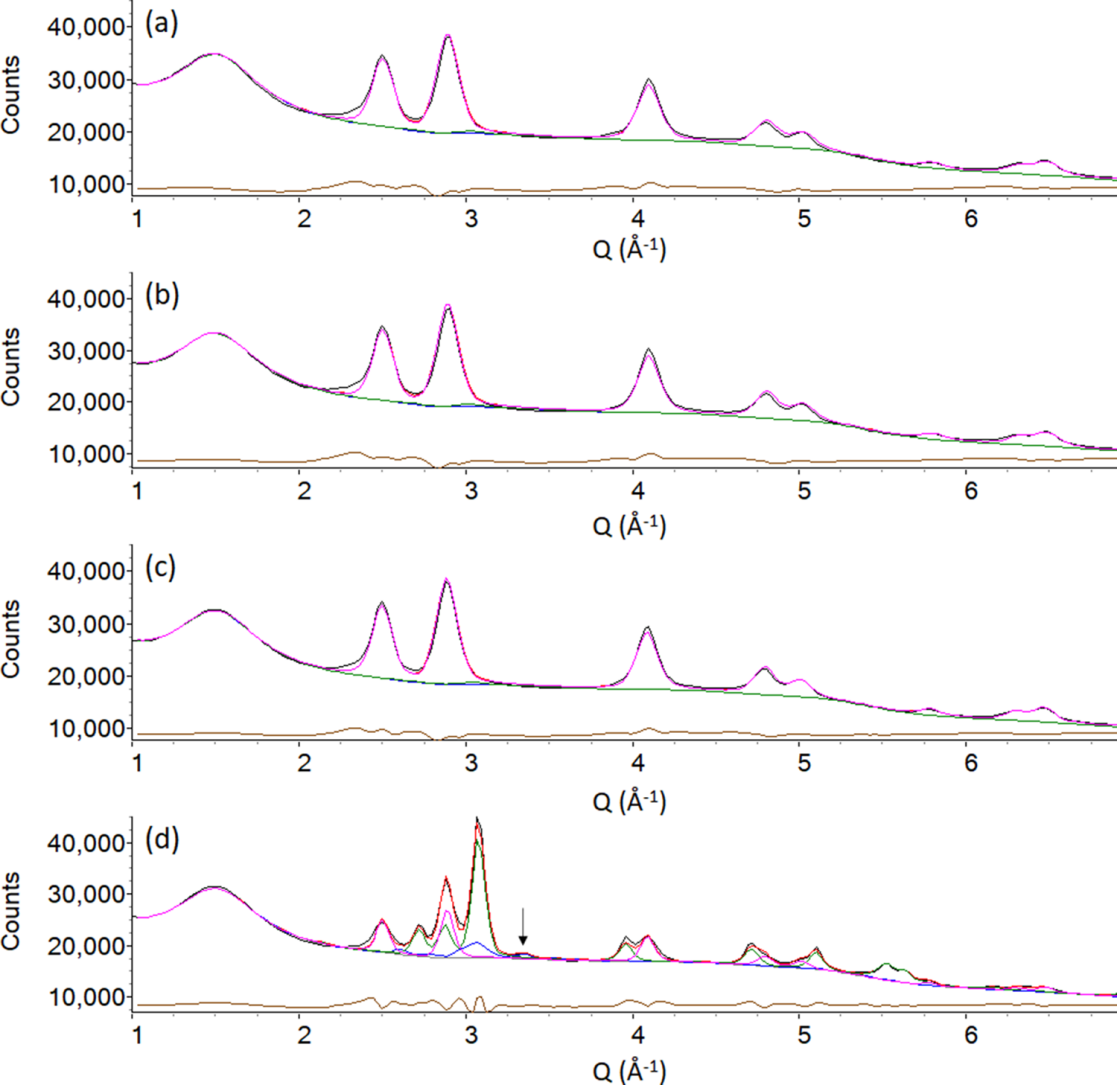
Rietveld plots from the *in situ* Fe(NO_3_)_3_/gelatin sol–gel reaction, (a) *T* = 350 °C, *R*_wp_ = 1.605%; (b) *T* = 400 °C, *R*_wp_ = 1.729%; (c) *T* = 500 °C, *R*_wp_ = 1.382%; and (d) *T* = 600 °C, *R*_wp_ = 1.602%. The black curves represent the observed data, the red curves the total calculated pattern, the brown curves are the difference between observed and calculated pattern, the pink curves the calculated pattern arising from FeO*_x_*, the green curves the calculated pattern arising from Fe_3_N, the blue curves represent the pattern arising from Fe_3_C, and the gray curves are the background. In panels (a–c), Fe_3_C and Fe_3_N were included in the refinements to ensure trace amounts were not missed but contribute 0% to the calculated pattern. The peak in panel (d) highlighted with the black arrow arises from Fe_3_C.

The data from 200 to 300 °C produced negligible Bragg scattering and so were fitted using only the empty capillary background (Figure S1). For data collected from 350 to 600 °C, Rietveld analysis was used in order to estimate the compositions of crystalline components to produce the PDF data. Figure 2, showing the observed data and Rietveld plots, shows that for 350 ≤ *T* ≤ 500 °C, there is a very little change in the composition of the sample. FeO*_x_* (wüstite) is the only crystalline phase present and the broad peaks (indicative of a small crystallite size) are consistent with the previous synchrotron diffraction data.^12^ At *T* = 600 °C, there is a dramatic change in the pattern where Fe_3_N becomes the major phase, with some FeO*_x_* still present and Fe_3_C beginning to form. Several peaks that arise from Fe_3_C are in similar positions to those that are from Fe_3_N, which can potentially result in Rietveld refinement software fitting the background using the structural parameters of Fe_3_C. Despite the weak intensity arising from Fe_3_C, there is evidence to suggest the presence of crystalline Fe_3_C. The peak at *Q* = 3.3 Å^–1^ arises from Fe_3_C and is unaccounted for in refinements where Fe_3_C was excluded. Additionally, our previous *in situ* synchrotron diffraction experiment,^12^ which was performed using a high Bragg resolution instrument, also showed Fe_3_C beginning to form at 600 °C. The calculated weight percentages for FeO*_x_*, Fe_3_N, and Fe_3_C are approximately 29, 48, and 22%, respectively. It is likely that the calculated weight percentage for Fe_3_C is higher than the real weight percentage. The “excess” Fe_3_C that is calculated here is calculated at the expense of Fe_3_N due to the large overlap of potential Bragg peaks. However, for the purpose of this study, which was to obtain an approximate composition for processing the PDF data, the effect is negligible. This is due to the very similar X-ray scattering lengths and densities of Fe_3_N and Fe_3_C. The peaks arising from Fe_3_N are also far more noticeable than those from Fe_3_C due to the higher symmetry of Fe_3_N (*P*6_3_22 compared to *Pnma*). The peak sharpness also indicates that the Fe_3_N phase is more crystalline and has bigger particles than the FeO*_x_* phase.^28,29^ The synchrotron XRD experiment showed Fe_3_O_4_ at low temperatures, which is not observed in this new data, presumably due to the longer scan times (i.e., periods of thermal equilibrium) required for collecting data of sufficient quality for PDF analysis. Indeed, our previous laboratory studies have also shown that the onset point of the Fe_3_O_4_ to FeO*_x_* transition is dependent on experimental conditions.^13^ It is also possible that the experimental setup (N_2_ gas flowing over the end of a closed capillary as opposed to through the capillary via a retort) caused this slight variation in the system.

As Bragg scattering can only be produced by materials with a long-range order (i.e., crystalline materials), it cannot tell us about the nature of any amorphous phases present. However, as carbon is likely to make up most of the amorphous fraction of the sample, the contribution of the amorphous phases to the total scattering factor will be negligible compared to the much more electron-dense crystalline iron compounds. Therefore, the phase compositions extracted from the Rietveld refinements represent a good approximation for use in the total scattering processing to produce the PDFs.

### 3.2 PDF Analysis

**Figure 3:**
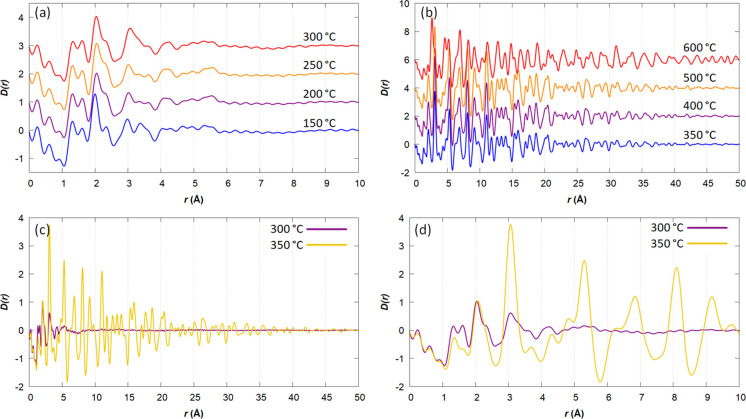
PDFs of the Fe(NO_3_)_3_/gelatin sol–gel reaction obtained at (a) *T* = 150–300 °C, (b) *T* = 350–600 °C, and *T* = 300 °C vs 350 °C from (c) 0–50 Å and (d) 0–10 Å.

In order to probe the local structure, the PDFs from 150 to 600 °C were produced. Due to the similar peak positions and X-ray scattering of Fe_3_C and Fe_3_N, we also processed the data using Rietveld refinements where Fe_3_C was excluded. The resultant PDFs at 600 °C were nearly identical (Figure S2), indicating that even if the Fe_3_C composition has been overestimated this will not impact the conclusions from the PDF analysis. Figure 3a shows the PDFs obtained for samples from 150 to 300 °C. The data illustrate that the iron oxide phase, which is most likely to be Fe_3_O_4_ at this temperature, based on previous studies^12^ has a very short-range order with no correlations above r = 6 Å. This is an even shorter range of order than is observed in amorphous carbon, which typically shows correlations in the range of 10 ≤ *r* ≤ 20 Å.^30,31^ Fe_3_O_4_ has been shown to produce highly crystalline nanoparticles when synthesized via a solution route, with correlations extending nearly through the entire nanoparticle.^32^ The very short-range order in our system therefore suggests that the sample is completely amorphous up to 300 °C rather than containing very small crystalline iron oxide nucleation clusters. Sol–gel methods have for many years been promoted as routes that maximize homogeneity in solid-state precursors^33^ and these PDF data are direct evidence that this is in fact the case.

Between 150 and 200 °C, there is a peak in the region of 2.0 ≤ *r* ≤ 2.6 Å that shifts and broadens. This region corresponds to the average Fe–O distances in Fe_3_O_4_ (1.89 and 2.06 Å) and FeO*_x_* (2.16 Å).^19,34^ and the shift in the large peak from ∼2.0 to ∼2.1 Å suggests the carbothermal reduction of amorphous Fe_3_O_4_ to FeO*_x_*. It is possible that the shift is due to the final decomposition of the iron nitrate precursor. However, there is also a further peak broadening from 250 to 300 °C at 3.0 ≤ *r* ≤ 3.8 Å, corresponding to the first Fe–Fe distance in Fe_3_O_4_ and FeO*_x_*, which provides further evidence that an amorphous Fe_3_O_4_ phase is being converted to FeO*_x_*.

Figure 3b shows the PDFs in the temperature range of 350–600 °C, where the samples show Bragg scattering. The first thing that should be noted is the difference in scale. There is a dramatic change from 300 to 350 °C in a system that has a much higher range of order, with correlations that extend to >40 Å. This is highlighted in Figure 3c which shows the fast (5 min) transition from an amorphous material with a very short-range order at 300 °C to a material with clear crystalline regions at 350 °C. The transition is consistent with the emergence of Bragg peaks for FeO*_x_*, showing the onset of crystallization of FeO*_x_* nanoparticles from the amorphous precursor. The local structures at 300 and 350 °C are similar at <5 Å (Figure 3d), though the peaks are a lot sharper at 350 °C. This indicates that the change in the system is a structural rearrangement of the locally disordered iron oxide material into ordered domains rather than a chemical transition as there are atom pairs distributed at very similar values. The broader distributions found at 300 °C are likely due to the increased disorder compared to those at 350 °C, though there is a possibility of a small quantity of left over precursor materials. This reflects the similar observations that have been made in solution-state crystallization processes, such as the formation of amorphous NaCl clusters followed by a sudden onset of crystallization.^35^

An attempt was made to refine the local structure at 350 °C using FeO*_x_* and an amorphous carbon phase. In this case, we used a refinement with FeO*_x_* using its long-range symmetry (*Fm*-3*m*) and a graphite phase with correlations at *r* >5 Å removed. This was found to provide a better fit (Figure S3a, *R*_wp_ = 22.029% and χ^2^ = 0.173) than with short-range diamond or a diamond/graphite combination. This fit was not satisfactory for *r* <4 Å, so another phase of FeO*_x_* with a lower *P*4 symmetry was added to account for local breaking of symmetry (Figure S3b). Having a lower symmetry in the local structure while having a higher symmetry long-range structure due to disorder is commonly observed in oxide materials such as Ba_2_In_2_O_5_ and La_2_Mo_2_O_9_.^36,37^ While this did improve the fit (*R*_wp_ = 19.150% and χ^2^ = 0.151), there were still some discrepancies with the peak at *r* = 1.4 Å, corresponding to the carbon phases, and the peak at *r* = 2.1 Å, corresponding to the nearest neighbor Fe–O distance. FeO*_x_* is known to have a highly defective (and often oxygen-deficient) structure containing Frenkel defects.^19^ This fact, combined with the complexity of the overall system, means that there could be many factors contributing to peak broadening and shifting.

From 350 to 500 °C, there is a very little change in the PDF, suggesting that there is no significant growth in the FeO*_x_* nanoparticles. Between 500 and 600 °C, there is another dramatic change, and in this case, there is a substantial shift in peak intensities and positions, correlating to the observation of Fe_3_N peaks in the Bragg scattering. At 600 °C, there are correlations up to *r* = 50 Å, which is the maximum distance that the PDFs were processed to. This indicates a growth in the crystallite size during the FeO*_x_* to Fe_3_N transition.

**Figure 4:**
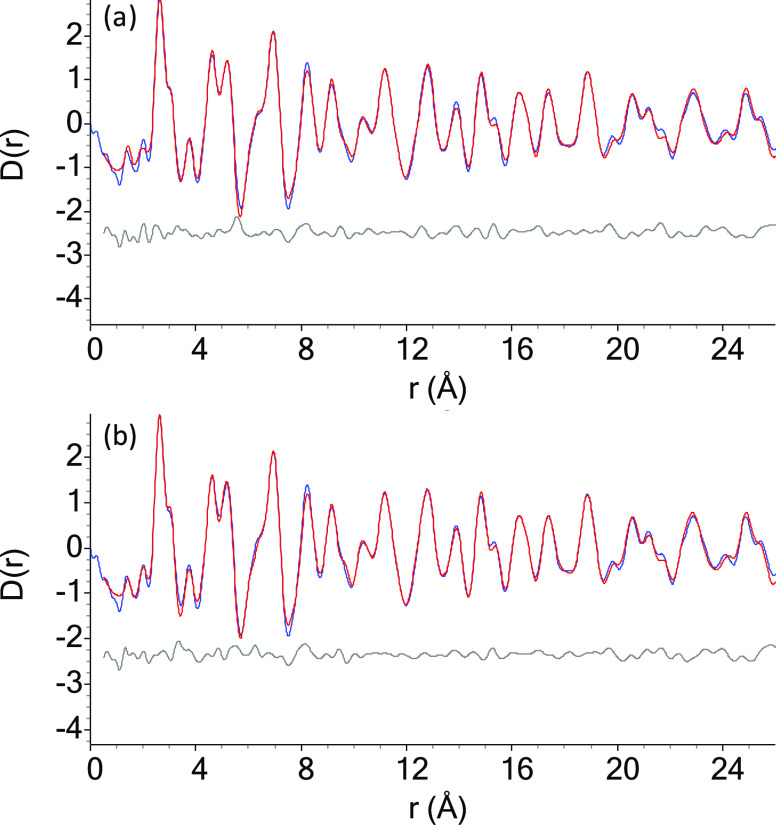
PDF refinements of the Fe(NO_3_)_3_/gelatin sol–gel reaction at 600 °C. (a) Refinement with FeO*_x_*, Fe_3_C, amorphous sp^2^ and sp^3^ carbon phases, and only *P*6_3_22 Fe_3_N, *R*_wp_ = 12.318%, and χ^2^ = 0.099; (b) refinement with FeO*_x_*, Fe_3_C, amorphous sp^2^ and sp^3^ carbon phases, and two phases of Fe_3_N: one with *P*6_3_ symmetry for *r* <5.0 Å and one with *P*6_3_22 for *r* >5.0 Å, *R*_wp_ = 11.629%, and χ^2^ = 0.094. Blue curve = observed PDF and red curve = calculated PDF.

In order to fully characterize the PDF data for the complex mixture of components present at 600 °C, structural refinements were performed for Fe_3_C, Fe_3_N, and FeO*_x_* using TOPAS. The raw data and the resulting fit are shown in Figure 4. One challenge with the analysis was how to reasonably include the amorphous carbon component. TOPAS v6 can only include crystalline phases;^22^ however, it also permits the use of functions that scale calculated contributions as an arbitrary function of distance. This allows amorphous phases with a very short-range order to be approximated. Therefore, both diamond and graphite were included in our refinement (with correlations for *r* >5 Å removed) to model the mixture of sp^2^ and sp^3^ carbons that result from the decomposition of gelatin.^38^ The C–C distances found in sp^2^ and sp^3^ carbons range from 1.4 to 1.5 Å, corresponding to the first peak in the PDF at ∼1.42 Å. As the majority of the scattering is produced by the Fe-containing phases and the primary purpose of this study is the structure of the Fe_3_N nanoparticles, we propose that this approximation for the carbon phase is sufficient. The cell parameters of all included phases were refined according to their long-range symmetry as well as spherical atomic displacement parameters. Atomic coordinates were only refined for Fe_3_N as it was the primary phase, whereas Fe_3_C constituted only an estimated 22% by weight of the sample at this temperature, so there was not enough sensitivity in the PDF to accurately refine the coordinates.

Initial refinements used a single phase of Fe_3_N with its long-range space group of *P*6_3_22. While this resulted in a near-satisfactory fit (Figure 4a, *R*_wp_ = 12.318% and χ^2^ = 0.099), the first two peaks, corresponding to the amorphous carbon phases and the nearest-neighbor Fe–N distance in Fe_3_N, do not fit. They were instead shifted as a result of incorrect cell parameters. It is not uncommon for materials to locally break symmetry, such as in Ba_2_In_2_O_5_ and La_2_Mo_2_O_9_.^36,37^ In order to address this, a lower-symmetry *P*6_3_ phase of Fe_3_N was used in addition to the *P*6_3_22 phase. The *P*6_3_ space group is a maximal subgroup of *P*6_3_22, where the twofold rotational axes parallel and perpendicular to the *x* and *y* axes have been removed. This provides more degrees of freedom to the Fe atoms as they move from the 6*g* Wyckoff position [(*x*, 0, 0)] in the *P*6_3_22 phase to the general 6*c* Wyckoff position [(*x*, *y*, *z*)]. It also provides additional degrees of freedom to the N atoms as the *z* coordinate is allowed to be refined in the *P*6_3_ phase (Tables 1 and 2). By refining with just the *P*6_3_ phase, it was found that the optimal range for *r* to refine with the lower symmetry phase is *r* <5.0 Å. Thus, another refinement was performed including two phases of Fe_3_N: a *P*6_3_ phase contributing to the scattering at *r* <5.0 Å and a *P*6_3_22 phase contributing to the pattern at *r* >5.0 Å. This fit is shown in Figure 4b and resulted in better fitting of the positions of the first two peaks at *r* = 1.4 Å and *r* = 2.0 Å (*R*_wp_ = 11.629% and χ^2^ = 0.094). There is some intensity in the calculated curve at *r* < *r*_min_; this is due to TOPAS broadening the peak shape function.

**Table 1:** Structural Parameters of Fe_3_N at 600 °C in the Space Group *P*6_3_22^a^

site label	Wyckoff site	*x*	*y*	*z*	occupancy
Fe1	6*g*	0.336(3)	0	0	1
N1	2*c*	^1^/_3_	^2^/_3_	^1^/_4_	1

a Cell parameters: *a* = 4.6271(7) Å, *c* = 4.3664(9) Å, α = 90°, γ = 120°, and *V* = 80.96(3) Å^3^.

**Table 2:** Structural Parameters of Fe_3_N at 600 °C in the Space Group *P*6_3_^a^

site label	Wyckoff site	*x*	*y*	*z*	occupancy
Fe1	6*c*	0.326(3)	0.045(3)	0(2)	1
N1	2*b*	^1^/_3_	^2^/_3_	0.2(2)	1

a Cell parameters: *a* = 4.72(1) Å, *c* = 4.48(2) Å, α = 90°, γ = 120°, and *V* = 86.3(5) Å^3^.

**Figure 5:**
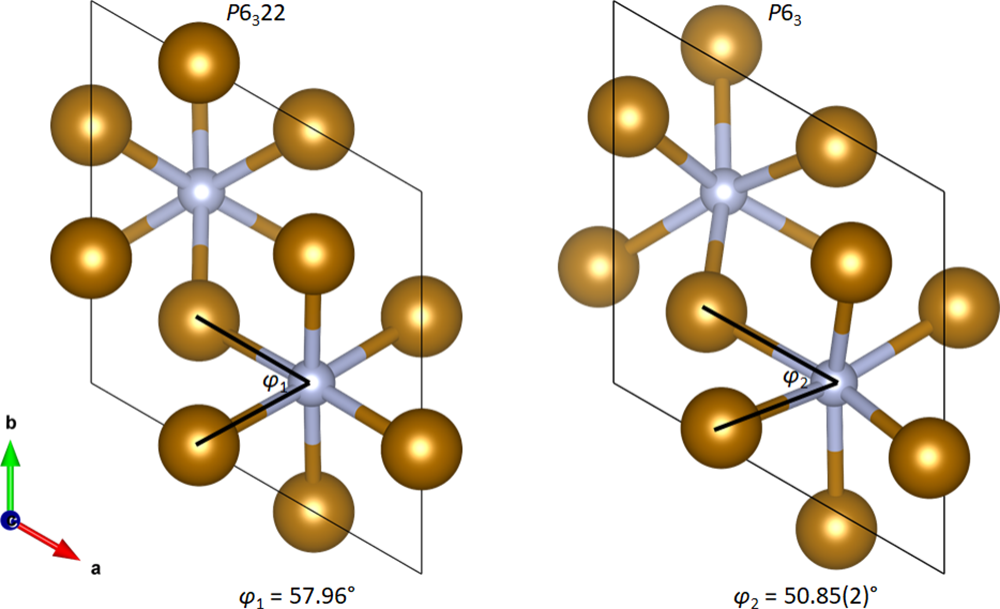
Twist angles obtained from Fe_3_N in *P*6_3_22 at room temperature^21^ and in *P*6_3_ at 600 °C. Fe atoms are shown in gold and N in silver. The figure is a 2D projection and the angles shown do not include the *z* coordinates of the atoms.

Due to the increased degrees of freedom of the Fe and N atoms, the twist angle of the NFe_6_ octahedra is adjusted in the *P*6_3_ phase. The twist angle, φ, is a parameter used in coordination chemistry to describe how trigonal-prismatic or octahedral in nature a sixfold coordinate polyhedron is, where φ = 0° is a perfect trigonal prism and φ = 60° is a perfect octahedron.^39,40^ In the long-range order, the average structure of Fe_3_N with a *P*6_3_22 symmetry, φ_1_ = 57.96°,^21^ is an almost perfect octahedron. The CFe_6_ polyhedra in Fe_3_C are trigonal prisms, so a gradual shift in the local structure could be expected if the nitride to carbide transformation occurs via gradual replacement of N atoms with C.^41,42^ The twist angle in the *P*6_3_ phase in our system was found to be φ_2_ = 50.85(2)°. The difference between the two structures is illustrated in Figure 5. While the conformation is still primarily octahedral, the NFe_6_ polyhedra are distorted and more trigonal prismatic in nature compared to the average structure. Given that iron carbonitride phases are known to exist,^12,43^ it is plausible that the distortion in the octahedra could be due to the incorporation of C into the structure as Fe_3_N reacts with the surrounding carbon during the formation of Fe_3_C. A detailed *ex situ* total scattering study of these systems would be necessary to establish whether the distortion is indeed due to carbon diffusion or whether it is an intrinsic feature of Fe_3_N. Distinguishing between carbon and nitrogen through atomic form factors alone is challenging in total scattering but is possible by comparing the bond lengths.

## 4 Conclusions

*In situ* total scattering has been used to probe the evolution of FeO*_x_* and Fe_3_N nanoparticles from a Fe(NO_3_)_3_/gelatin sol–gel precursor. Despite the complex, multicomponent nature of the system, we were able to extract valuable information about the intermediate phases and phase transitions. The onset of crystallization is very fast. Correlations in the PDFs are only observed at <6 Å at 300 °C, indicating a highly amorphous structure. At 350 °C, however, there are correlations up to ∼40 Å and this is only 5 min further on in the synthesis. This lack of change in the short-range order during this transition indicates that crystallization of FeO*_x_* nanoparticles occurs from the local structural rearrangement of the atoms. Given that the size and nature of intermediate oxide phases in the sol–gel synthesis can dramatically affect the nature and morphology of a ceramic product,^44^ this ability to observe early nucleation stages *in situ* could enable us to tune the synthesis conditions in our system to achieve more control over the particle size. Our results also offer insight into the Fe_3_N phase. At 600 °C, when Fe_3_N becomes the dominant phase, the nanoparticles have a longer-range order, suggesting a larger particle size. Structural refinements reveal that the NFe_6_ octahedra present in the Fe_3_N phase at 600 °C are in fact distorted, resulting in symmetry lowering in the local structure from *P*6_3_22 to *P*6_3_. The distortions to the NFe_6_ octahedra may be caused by carbon beginning to replace N within the Fe lattice as the structure of Fe_3_C consists of CFe_6_ trigonal metaprisms. In summary, the study has offered us a unique insight into the mechanism of Fe_3_C nanoparticle formation by sol–gel chemistry. Given that controlling the particle size is very important in metal carbide chemistry, these results suggest that focusing on the distribution of amorphous metal oxides in the precursor material will be crucial in reducing the particle size of the final carbide.

## Supporting Information Available

Rietveld refinements of data <300 °C, PDF refinements of 350 °C samples, and structural parameters of FeO*_x_* and Fe_3_C (PDF)
