# Supplementary material for: Evolution of the Local Structure in the Sol–Gel Synthesis of Fe3C Nanostructures
Source: Inorg Chem. 2021 May 4;60(10):7062–9. doi: 10.1021/acs.inorgchem.0c03692 (PMC8277138; doi:10.1021/acs.inorgchem.0c03692)
Supplement: Supplementary file 1 — ic0c03692_si_001.pdf [file ic0c03692_si_001.pdf]

Evolution of the Local Structure in the Sol-Gel Synthesis of Fe_3C Nanostructures: Supporting Information

Matthew S. Chambers,[*,†] Dean S. Keeble,[‡] Dean Fletcher,[†] Joseph A. Hriljac[†,‡] and Zoe Schnepp[*,†]

†School of Chemistry, University of Birmingham, Birmingham, B152TT, United Kingdom

‡Diamond Light Source, Harwell Science and Innovation Campus, Didcot, OX11 0DE, United Kingdom

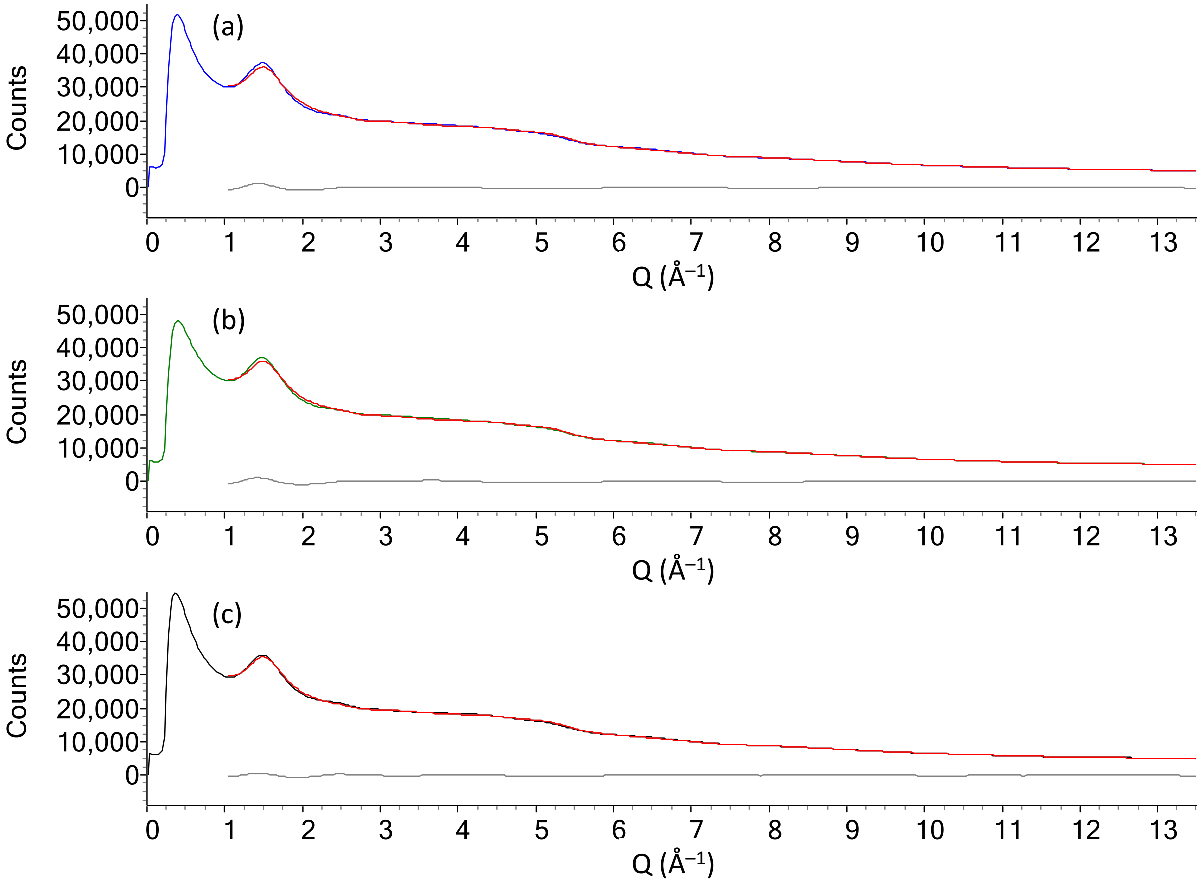

Figure S1 – Rietveld plots from the *in-situ* $Fe(NO_3)_3$/gelatin sol-gel reaction at *T* < 350 °C; (a) 200 °C; (b) 250 °C; (c) 300 °C. The blue, black and green curves represent the observed data, the red curves represent the calculated pattern and the grey curves represent the difference. In all three plots, only the empty capillary background curve was included; no phase information was included, indicating that the Bragg scattering present was caused by the silica capillary.

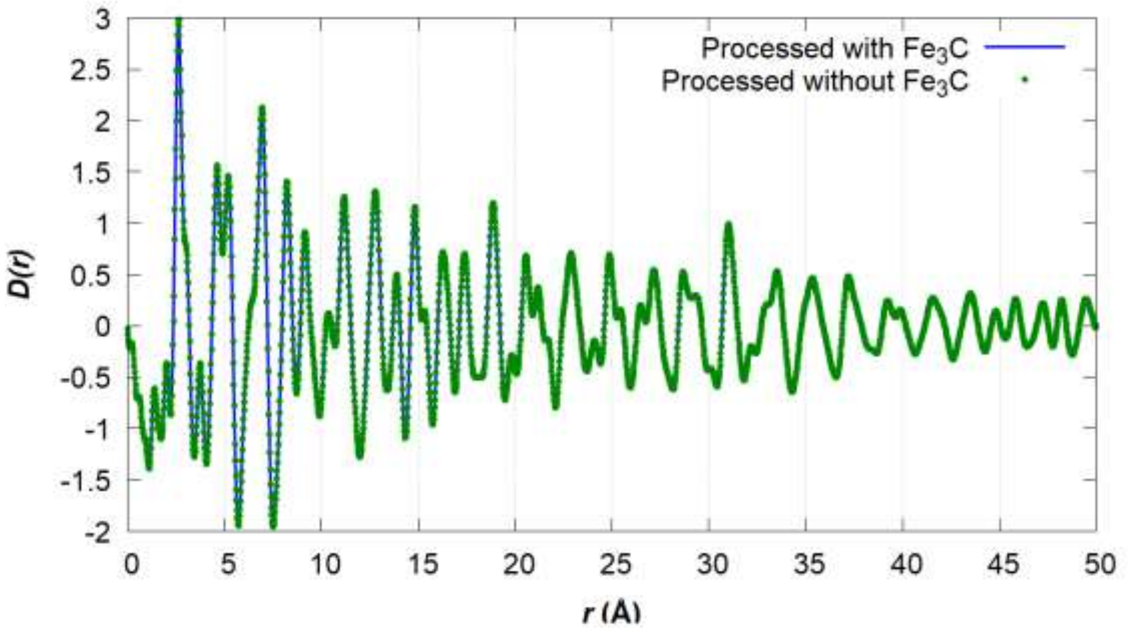

Figure S2 The PDFs of $Fe(NO_3)_3$/gelatin sol-gel reaction obtained at 600 °C produced using the elemental compositions obtained from Rietveld refinements including Fe_3C and excluding Fe_3C.

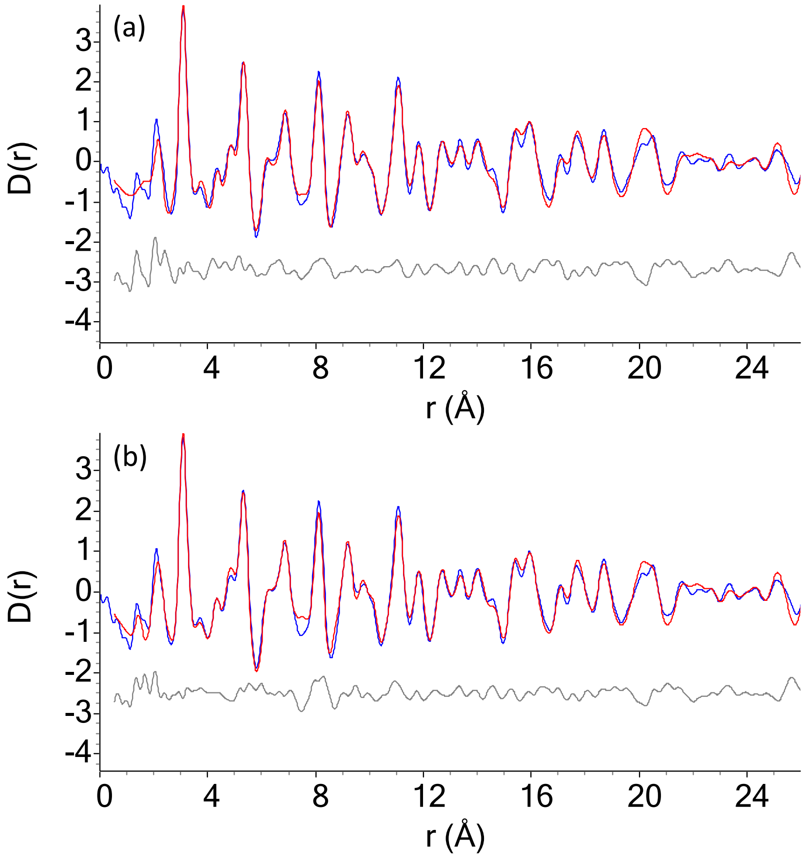

Figure S3 The PDF refinements of $Fe(NO_3)_3$/gelatin sol-gel reaction at 350 °C. (a) Refinement with sp^2 carbon phase and only *Fm*-3*m* FeO_x, R_{wp} = 22.029%, χ^2 = 0.173; (b) refinement with sp^2 carbon phase, *P*4 FeO_x at $r < 5$ Å and *Fm*-3*m* FeO_x at $r > 5$ Å, R_{wp} = 19.150%, χ^2 = 0.151%. Blue curves are the observed PDFs, red curves are the calculated PDFs.

Table S1 – The structural parameters of FeO_x at 350 °C in the space group *Fm*-3*m*. Cell parameters: *a* = 4.3397(5) Å, *α* = 90°, *V* = 81.73(3) Å^3

Site label	Wyckoff Site	*x*	*y*	*z*	Occupancy
Fe1	4*a*	0	0	0	1
O1	4*b*	$^1/_2$	$^1/_2$	$^1/_2$	1

Table S2 – The structural parameters of FeO_x at 350 °C in the space group *P*4. Cell parameters: *a* = 4.3397(5) Å, *α* = 90°, *V* = 81.73(3) Å^3

Site label	Wyckoff Site	*x*	*y*	*z*	Occupancy
Fe1_1	1*a*	0	0	0.0(7)	1
Fe1_2	2*c*	$^1/_2$	$^1/_2$	0.4	1
Fe1_3	1*b*	$^1/_2$	$^1/_2$	0.0(7)	1
O1_1	1*b*	$^1/_2$	$^1/_2$	0.4(7)	1
O1_2	2*c*	$^1/_2$	0	-0.09(3)	1
O1_3	1*a*	0	0	0.4(7)	1

Table S3 – The structural parameters of Fe_3C at 600 °C. Space group = *Pnma*; cell parameters: *a* = 5.04(1) Å, *b* = 6.88(1) Å, *c* = 4.585(8) Å, *α* = 90°, *V* = 159.2(5) Å^3

Site label	Wyckoff Site	*x*	*y*	*z*	Occupancy
Fe1	8*d*	0.1846	0.0594	0.3340	1
Fe2	4*c*	0.0346	$^1/_4$	0.8377	1
C1	4*c*	0.8984	$^{1/4}$	0.4467	1
